# Mechanism of semen liquefaction and its potential for a novel non-hormonal contraception^†^

**DOI:** 10.1093/biolre/ioaa075

**Published:** 2020-05-14

**Authors:** Prashanth Anamthathmakula, Wipawee Winuthayanon

**Affiliations:** School of Molecular Biosciences, Center for Reproductive Biology, College of Veterinary Medicine, Washington State University, Pullman, WA 99164, USA

**Keywords:** semen liquefaction, semenogelins, kallikrein-related peptidase, prostate-specific antigen, sperm motility, contraceptive, fertility

## Abstract

Semen liquefaction is a proteolytic process where a gel-like ejaculated semen becomes watery due to the enzymatic activity of prostate-derived serine proteases in the female reproductive tract. The liquefaction process is crucial for the sperm to gain their motility and successful transport to the fertilization site in Fallopian tubes (or oviducts in animals). Hyperviscous semen or failure in liquefaction is one of the causes of male infertility. Therefore, the biochemical inhibition of serine proteases in the female reproductive tract after ejaculation is a prime target for novel contraceptive development. Herein, we will discuss protein components in the ejaculates responsible for semen liquefaction and any developments of contraceptive methods in the past that involve the liquefaction process.

## Teaser

We propose inhibition of semen liquefaction has the potential to be developed as a non-hormonal contraceptive method.

## Introduction

The fate of ejaculated spermatozoa in humans is very different from that in rodents. Male mice and rats ejaculate sperm and accessory gland secretions (e.g., seminal vesicle, prostate) directly into the uterus and produce a copulatory plug, which is not liquefied in vivo. In humans, however, the ejaculate is deposited in the anterior wall of the vagina, which later liquefies, and the sperm gain their motility to transport to the upper female reproductive tract for fertilization (reviewed in [1]).

**Figure 1:**
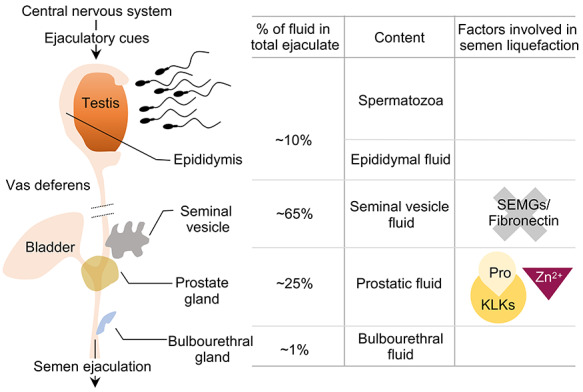
Fluid components in human ejaculate. The majority of semen is made up of seminal vesicle fluid (~65%; containing semenolgelins or SEMGs and fibronectin) and prostatic fluid (~25%; containing pro-kallikrein (Pro-KLK) enzymes and Zn^2+^). Epididymal fluid and testis make up to ~10% of the semen, while bulbourethral gland (mostly secretes mucinous proteins) is only 1%.

In humans, the semen is a fluid conglomerate consisting of two major components: the cellular fraction (consisting of spermatozoa, migrating leucocytes, immature germ cells, and epithelial cells) and acellular fraction consisting of seminal plasma and extracellular vesicles (epididymosomes and prostasomes) (Figure 1) (reviewed in [2]). Human semen consists of approximately 2–5% spermatozoa and 98–95% seminal plasma, have a minimum volume of 2 mL, a pH of 7.2–8.0 and contain 200–500 million spermatozoa. The liquefaction process requires proteins present in the acellular fraction (seminal plasma) of the semen. Therefore, before describing the process, we will discuss necessary protein components present in the seminal plasma that are involved in the liquefaction process.

## Seminal plasma

The seminal plasma is rich in sugars, glycans, lipids, inorganic ions, metabolites, cell-free DNA, microRNAs, peptides, and proteins, which are secreted from seminal vesicles, prostate, epididymis, and bulbourethral glands (reviewed in [3]). Seminal vesicles contribute to ~65% of the semen volume and are rich in semenogelins (SEMGs), fibronectin, prostaglandins, cytokines, and fructose, while the prostatic secretions are rich in proteolytic enzymes, citrate, and lipids and contribute to ~25% of the total volume of seminal fluid (Figure 1). The semen has an alkaline pH (7.2–8.0) from seminal vesicles and prostate secretions containing basic polyamines such as spermine, spermidine, and putrescine, which counteract the vaginal acidity and are important for sperm survival. Secretions from bulbourethral glands (contain mucins, galactose, sialic acid) contribute to ~1% of semen volume and act as lubricants enabling efficient sperm transfer (reviewed in [2]). The seminal plasma proteins play an important role in semen coagulation, sperm motility, capacitation, acrosome reaction, and immune activity suppression in the female reproductive tract (reviewed in [3]). Here, we will discuss the function of key factors present in the seminal plasma that are involved in the semen liquefaction process.

### Seminal vesicle secretions: SEMGs

Semenogelin proteins (encoded by *SEMG1* and *SEMG2* genes) are secreted from seminal vesicles [4]. SEMG1 and SEMG2 are the two major proteins of the seminal coagulum and represent 20–40% of the seminal plasma proteins [5, 6]. SEMG1, a predominant 52 kDa protein, contains a single cysteine residue at position 239 (Cys^239^) and forms intermolecular disulfide bridges with the less abundant SEMG2 (exist as non-glycosylated 71 kDa and glycosylated 76 kDa) at Cys^159^ and Cys^360^ residues, resulting in high molecular weight complex SEMGs (reviewed in [7]). Upon ejaculation, semen immediately turns into a gelatinous meshwork of crosslinking SEMGs. As a result, sperm are entrapped within the seminal coagulum. The N-terminal fragment of SEMG1 was originally identified as the region of seminal plasma motility inhibitor (SPMI) [8, 9]. In addition, the C-terminal fragment of SEMG1 containing Cys^239^ (164–283 amino acids) was found to have significant inhibitory effects on both motility and progressive motility of intact live human spermatozoa [10]. Accordingly, O’Rand et al. [11] reported that recombinant human SEMG inhibits sperm progressive motility. In this context, a study by Yamakasi et al. [12] also indicated that patients with higher number of SEMG-unbound spermatozoa can achieve successful pregnancy, making total SEMG-unbound sperm count a relevant parameter for in vivo fertilization. Additionally, SEMG peptides are also involved in other biological functions such as increasing sperm hyaluronidase activity [13], antibacterial activity [14], hyperpolarization, and permeability of sperm plasma membrane [15].

### Prostatic fluid: kallikreins

Prostate-specific antigen (also known as kallikrein-related peptidase 3 or KLK3), prostatic acid phosphatase, and prostate secretory protein of 94 amino acids (PSP94) are the three predominant proteins in the prostate fluid secreted by the prostate gland [16]. Tissue kallikrein-related peptidases (KLKs) are trypsin- and/or chymotrypsin-like serine proteases secreted by the prostatic epithelial cells. The KLK locus, the largest contiguous cluster of serine proteases, is localized on human chromosome 19 and encodes *KLK1-15*. Despite 36–77% homology among the 15 KLKs at the protein level, amino acid sequences surrounding the catalytic triad (His^57^, Asp^102^, and Ser^195^) are highly conserved among mammalian species (reviewed in [17]). Tissue KLKs are different from plasma kallikrein, which is a liver-derived protease, encoded by *KLKB1* gene on human chromosome 4 [18]. Shaw and Diamandis [19] performed a comprehensive expression profiling of KLKs in human tissues and biological fluids and observed that the majority of KLKs (KLK1–5, 9, 11, 13–15) are expressed by the prostate and secreted into seminal plasma.

**Figure 2:**
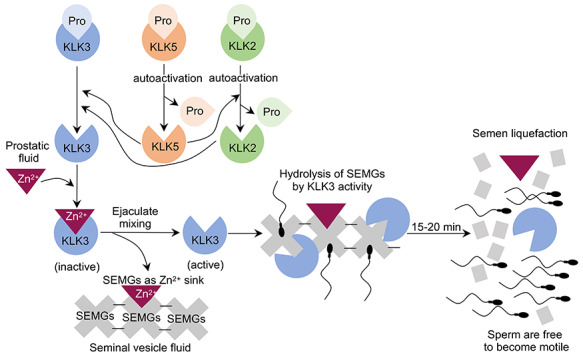
Signaling cascade of kallikrein 3 (KLK3) activation during liquefaction process. Pro-KLKs are secreted into the prostatic fluid. High concentration of Zn^2+^ in prostatic fluid inactivates KLK3 activity. After ejaculation, prostatic and seminal vesicle fluids are combined. SEMGs are available to sequester Zn^2+^ as SEMGs have higher affinity to Zn^2+^ compared to KLKs. Pro-KLK5 undergoes autocleavage to rid of pro-peptide sequences and autoactivates. Subsequently, KLK5 activates pro-KLK2 and 3. KLK2 also potentially activates pro-KLK3. Activated KLK3 then hydrolyzes SEMGs into smaller fractions. After hydrolysis, semen becomes liquefied and sperm gain their motility to transport to the upper female reproductive tract for fertilization.

KLKs are initially synthesized as pre-pro-KLK proteins, and then the pre-peptides are removed during secretion. Pro-KLKs are inactive, and extracellular cleavage of their amino-terminal pro-peptide by proteolytic activation cascades governs the semen liquefaction mechanism. After secretion, the zymogen activation cascade is initiated by pro-KLK5, which undergoes autoactivation [20] and subsequently activates downstream pro-KLK2, 3, 7, 8, and 14 [17, 21] (Figure 2). Activated KLK14, in turn, also activates pro-KLK5 in a positive feedback loop [20]. Active KLK2 is a known activator of pro-KLK3 [22–25] (Figure 2). Additional prostatic KLKs such as KLK4 [26] and KLK14 [27, 28] have also been reported to activate pro-KLK3 and presumably aid in semen liquefaction. KLK14 is also involved in the activation of pro-KLK1 and KLK11 [27]. While their role in semen liquefaction is not completely known, KLK11 is expressed in intermediate amounts in seminal plasma at concentrations ranging from 2 to 37 μg/mL [29], compared to other KLKs (detailed below). Interestingly, KLK2 and KLK3 are only present in the primates [30, 31] and have no known orthologs among the rodent KLKs.

In human body, prostate glands accumulate the highest levels of Zn^2+^ in prostatic epithelial cells in an androgen-dependent Zn^2+^ cellular uptake, which is mediated by specific zinc transporters [32]. The role of Zn^2+^ in liquefaction process will be discussed in the following section. In addition to KLKs, prostatic secretions are also rich in other proteins, such as prostatic acid phosphatase and zinc-α2-glycoprotein, which are involved in proteolytic cleavage of SEMGs [33], degradation of phospholipids [34], and lipid mobilization [35].

## Semen liquefaction process

**Figure 3:**
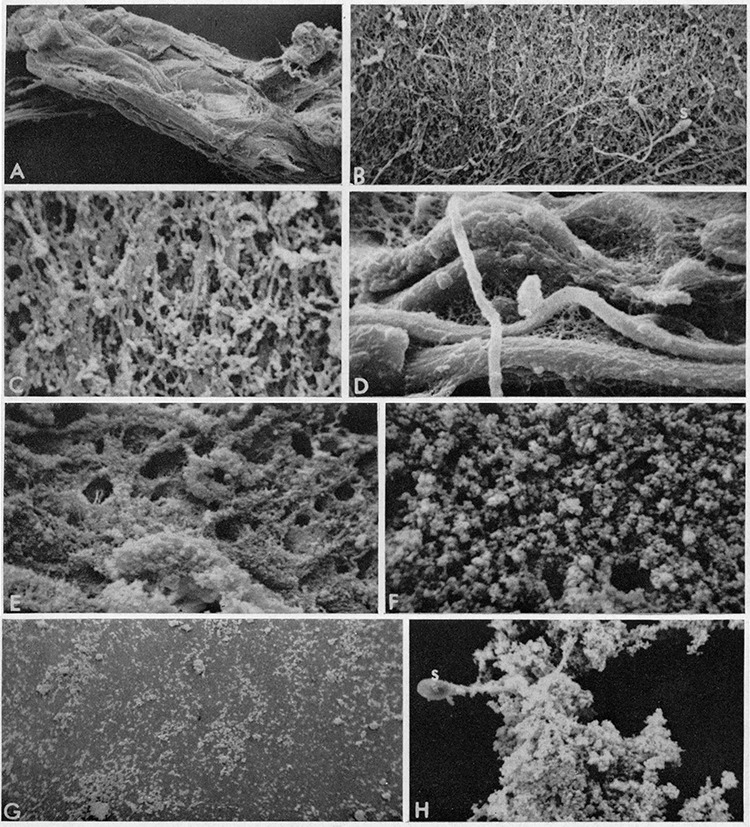
Scanning images of human semen before and after liquefaction. The samples were fixed at (A–D) 3 min, (E–F) 6 min, and (G–H) 15 min after ejaculation. Images in (G–H) were taken from samples immediately after liquefaction. (A) 30× magnification, (B) 600×, (C) 3000×, (D) 2875×, (E) 1200×, (F) 3100×, and (H) 1200×. S, spermatozoon. (A–C) and (E–H) normally liquefying; (D) slowly liquefying. Reprint of original images with permission from [37].

Semen liquefaction at the molecular level is characterized by progressive and site-specific cleavage of SEMGs into soluble low molecular weight proteins in the female reproductive tract [8, 36]. Human semen usually liquefies within ~15 to 20 min post-ejaculation [37] (Figure 3) and is a necessary step for further sperm processes related to fertilization, such as capacitation [38]. KLK3 is the major enzyme (staggering concentrations of 1290 μg/mL in seminal plasma [39]) that hydrolyzes SEMGs and fibronectin and liquefies semen coagulum facilitating sperm motility [8, 10, 36, 39–41]. KLK3 hydrolysis of SEMGs occurs preferentially at tyrosine, glutamine, and leucine and less commonly at other residues (histidine, aspartic acid, serine, and asparagine) [8, 41]. Other members of the KLK family participating in the process of semen liquefaction include KLK2 (concentration of 10–100 μg/mL), KLK5, and KLK14 (both enzymes ranging from 1 to 10 ng/mL) [19, 21, 23, 27, 42]. Active KLK2, 5, and 14 have been reported to cleave fibronectin and SEMGs in ex vivo and in vitro studies [21, 27, 28, 42–44]. Additionally, KLK6, 7, and 13 also exhibit catalytic activity toward fibronectin [45–47].

Semen liquefaction is also regulated by endogenous inhibitors such as Zn^2+^ (Figure 2) as well as protein C inhibitor (PCI). Prostatic KLKs are inactivated by allosteric reversible binding of Zn^2+^ in the seminal plasma (reviewed in [2]). Numerous studies have reported the ability of Zn^2+^ to inhibit KLK2 [43], KLK3 [8, 41, 48], KLK5 [21], and KLK14 [28] activities. Moreover, the inhibitory effect of Zn^2+^ on KLK3, 5, and 14 activities is reversible by SEMGs [21, 28, 48].

Once the ejaculation cue is triggered, SEMG-containing seminal vesicle secretions and prostatic fluid enriched with Zn^2+^ and KLKs are mixed with the sperm-enriched epididymal fluid to form a coagulum that entraps spermatozoa. Upon ejaculation, SEMGs sequester Zn^2+^ ions from KLKs as SEMGs possess a higher affinity to Zn^2+^, leading to KLK disinhibition and activation of the proteolytic cascade resulting in semen liquefaction (reviewed in [2]). Therefore, KLKs in concert with SEMGs regulate semen coagulation and liquefaction in a Zn^2+^-dependent manner. In addition to Zn^2+^, PCI has been shown to form a complex with SEMGs and KLKs to inhibit activities of KLKs in the seminal plasma [43, 49]. However, biological contribution of PCI in human semen liquefaction is widely unknown and requires further investigations.

## Factors affecting semen liquefaction

Genetic variations of genes involved in the liquefaction process as well as biochemical disruption could lead to liquefaction defect. This includes factors affecting the production and activity of KLKs, SEMGs, Zn^2+^, endogenous protease inhibitors, and other pathological conditions in male accessory organs (Table 1). As liquefaction process takes place in the female reproductive tract, local production of KLKs, endogenous protease inhibitors, and pathological conditions in the female tract could also be contributing factors for liquefaction defect. In clinical settings, the liquefaction time is of diagnostic importance if more than 1 h elapses without any change in the semen consistency [50]. Any defects in the liquefaction process can lead to impaired semen liquefaction and ~12% of infertility patients have the symptom of non-liquefied semen [51]. Here, we describe possible factors contributing to, or conditions resulting in defective semen liquefaction.

**Table 1:** Physiological functions of proteins in male accessory gland secretions potentially involved in regulating fertility in humans and rodents.

Protein	Gene (human)	Gene (mouse)	Function	Phenotypes when mutated, overexpressed, or genetically ablated
**Seminal vesicles**				
SEMG1	*SEMG1*	*Svs2*	SEMG1 forms intermolecular disulfide bridges with SEMG2 resulting in high molecular weight coagulum upon ejaculation [7]. Inhibits sperm motility [10, 11]. SVS2 is a known decapacitation factor and maintains sperm motility and sperm cholesterol levels, prevents spontaneous sperm capacitation, and is essential for sperm survival in the mouse uterus [66, 68]	*SEMG1* variants (rs147894843, rs2301366) associated with infertility [57, 63]. Elevated SEMG1 precursor reported in oligozoospermic men [64]. *Svs2*^−/−^ mice are subfertile with defects in copulatory plug formation [68]
SVS7	*SVS7*	*Pate4*	SVS7 in mouse is essential for copulatory plug formation in vivo [69]. SVS7 enhances mouse sperm motility in vitro [70]	No known mutation/phenotype reported in humans. *Pate4*^−/−^ mice are subfertile with defects in copulatory plug formation [69]
SERPINE2/PN1	*SERPINE2/PN1*	*Serpine2/Pn1*	Serine protease inhibitor acts as a decapacitation factor [82]. Inhibits protein tyrosine phosphorylation and sperm capacitation [82]	Elevated PN1 levels in semen of men displaying seminal dysfunction [85]. *Pn1^−/−^* mice are infertile due to altered seminal protein composition and defects in copulatory plug formation [85]
SPINK3/SPINK1	*SPINK3/SPINK1*	*Spink3/Spink1*	Serine protease inhibitor prevents premature acrosomal reaction and protects sperm in the uterine environment in mice [89, 90]	No known mutation/phenotype involving fertility reported in humans or mice
SPINKL	No known ortholog	*Spinkl*	Serine protease inhibitor acts as decapacitation factor and enhances sperm motility in mice [97]	No known mutation/phenotype involving fertility reported in humans or mice
**Testis and epididymis**				
EPPIN	*SPINLW1*	No known ortholog	Localized on the sperm surface. Modulates KLK3 activity and acts as decapacitating factor [10, 78, 79]. EPPIN-bound SEMG1 crucial for SEMGs degradation and initiation of progressive sperm motility [11]	*SPINLW1* upregulated in caput epididymis of non-obstructive azoospermic patients [80]. rs11594 variant associated with increased risk of idiopathic male infertility in Chinese–Han population [81].
**Epididymis**				
SPINK2	*SPINK2*	*Spink2*	Serine protease inhibitor protects sperm against protease activity during spermatogenesis	Homozygous *SPINK2* mutation leads to azoospermia in men [86]. Decreased *SPINK2* expression in azoospermic infertile men [87]. *Spink2* mutant mice have elevated serine protease activity and exhibit impaired fertility [88]. *Spink2^−/−^* mice are azoospermic and infertile [86]
SPINK5	*SPINK5*	*Spink5*	Serine protease inhibitor inhibits KLK5, 7, and 14 activities in corneocytes and regulates desquamation process [91, 92]	No known mutation/phenotype reported involving fertility in humans or mice
SPINK13	*SPINK13*	*Spink13*	Serine protease inhibitor. Essential for acrosomal integrity, sperm maturation, and fertility in rats [96]	No known mutation/phenotype reported in humans. *Spink13* knockdown rats demonstrate premature acrosomal reaction and reduced fertility [96]
**Prostate gland**				
KLK1	*KLK1*	*Klk1/mGK6*	**Serine protease**	Low level observed in SHV samples [58]. No known mutation/phenotype reported involving fertility in mice
KLK2/hK2	*KLK2*	No known ortholog	Serine protease cleaves fibronectin and SEMGs [42, 43]. Activator of pro-KLK3 [22–25]. Inhibited by Zn^2+^ [43]	Low KLK2 seminal levels observed in men with abnormal liquefaction and SHV [58]. SNP (rs2664155) associated with male infertility [61]
KLK3/PSA	*KLK3*	No known ortholog	Serine protease. Major enzyme hydrolyzes SEMGs and fibronectin and liquefies semen coagulum facilitating sperm motility [8, 10, 36, 39–41]. Inhibited by Zn^2+^ [8, 41, 48]	Low KLK3 level observed in men with SHV [55, 57] and abnormal liquefaction [58]. Reduced sperm motility observed in men with low seminal KLK3 levels [59]. SNPs (rs266881, rs174776, rs1810020, rs266875, rs35192866) associated with male infertility [60]
KLK4	*KLK4*	*Klk4*	Serine protease activates pro-KLK3 [26]	No known mutation/phenotype reported involving fertility in humans or mice
KLK5	*KLK5*	*Klk5*	Serine protease. Initiates liquefaction cascade by activating downstream pro-KLK2, 3, 7, 8 and 14 [17, 21]. Cleaves fibronectin and SEMGs [21, 44]. Inhibited by Zn^2+^ [21]	Low level observed in SHV samples [58]. No known mutation/phenotype reported involving fertility in mice
KLK6	*KLK6*	*Klk6*	Serine protease exhibits catalytic activity towards fibronectin [46]	Low level observed in SHV samples in humans [58]. No known mutation/phenotype reported involving fertility in mice
KLK7	*KLK7*	*Klk7*	Serine protease exhibits catalytic activity towards fibronectin [47]	*KLK7* (rs1654526) SNP associated with SHV in humans [57]. Low level observed in SHV samples in humans [58]. No known mutation/phenotype reported involving fertility in mice
KLK8	*KLK8*	*Klk8*	Serine protease	Low level observed in SHV samples [58]. No known mutation/phenotype reported involving fertility in mice
KLK10	*KLK10*	*Klk10*	**Serine protease**	Low level observed in SHV samples [58]. No known mutation/phenotype reported involving fertility in mice
KLK12	*KLK12*	*Klk12*	Serine protease	*KLK12* (rs61742847) SNP associated with SHV [57]. No known mutation/phenotype reported involving fertility in mice
KLK13	*KLK13*	*Klk13*	Serine protease exhibits catalytic activity towards fibronectin [44]	Low seminal levels observed in men with abnormal liquefaction and SHV [58]. No known mutation/phenotype reported involving fertility in mice
KLK14	*KLK14*	*Klk14*	Serine protease. Activates pro-KLK1, 3, 5 and 11 [20, 27, 28]. Cleaves fibronectin and SEMGs [27, 28]. Inhibited by Zn^2+^ [28]	Low seminal levels observed in men with clinically delayed liquefaction, SHV, and asthenospermia [28, 58]. KLK14 inhibition by ACT_G9_ delays semen liquefaction [27]. No known mutation/phenotype reported in mice involving fertility
TGM4	TGM4	*Tgm4*	A prostate-specific autoantigen plays a critical role in male reproduction and catalyzes the formation of N-ε-(γ-glutamyl)lysine cross-bridges between SEMGs in humans [72] and SVS proteins in mice [75], respectively	TGM4 autoantibodies are detected in subfertile adult male patients with autoimmune polyendocrine syndrome type 1, caused by mutations in autoimmune regulator (*AIRE*) gene [74]. *Tgm4*^−/−^ mice are subfertile with defects in copulatory plug formation and seminal fluid viscosity [75]. *Aire^−/−^* mice develop TGM4 autoantibodies, compromised TGM4 secretion, prostatitis, and exhibit subfertility [74].

### Semen hyperviscosity

According to WHO criteria, viscosity can be assessed in semen by observing the length of the thread formed by gently aspirating semen and allowing it to drop by gravity after 1-h incubation at room temperature [50]. A normal sample leaves the pipette in small discrete drops, while in cases of semen hyperviscosity (SHV), the drop will form a thread greater than 2 cm long [50]. Based on the thread length, SHV can be further classified into mild (2–4 cm), moderate (4–6 cm), and severe SHV (≥6 cm) [52].

SHV has a prevalence of 12–32% in men with fertility problems [52–54]. SHV negatively impacts semen quality and sperm motility because of the sperm-trapping effect of hyperviscous semen [53, 55]. Biochemical analysis of rheological properties of semen indicated the presence of highly organized peptide cores complexed with oligosaccharide chains and disulfide bonds in hyperviscous semen compared to normal samples [56]. Gopalkrishnan et al*.* [54] found that in semen samples with abnormal viscosity, the sperm count, motility, and chromatin integrity were significantly decreased when compared to controls with normal semen viscosity [54]. The etiology of SHV has often been attributed to male accessory gland infection, increased levels of leukocytes, and inflammation. Therefore, the composition of human seminal plasma is important in understanding the physiology of reproduction, and any alterations in seminal plasma may explain molecular mechanisms in some cases of infertility. The following section will discuss genetic variations of KLK enzymes that may contribute to SHV conditions in men and result in defective semen liquefaction.

### *KLK* mutations

Genetic factors may also influence the viscosity of seminal fluid. KLK3 level was significantly lower in SHV samples when compared to samples with normal viscosity [55, 57] suggesting the association between prostatic enzymes and semen viscosity. In a recent study, genetic variation within *KLK* locus was found to be associated with SHV [57]. *KLK7* (rs1654526) and *KLK12* (rs61742847) polymorphisms are significantly associated with SHV, while genetic variation in *KLK3* and *KLK15* was found to be three times higher in SHV samples than in controls [57]. Emami et al. [58] reported a possible role of KLKs in the pathogenesis of delayed semen liquefaction and SHV. Lower concentrations of KLK2, 3, 13, and 14 in men with abnormal liquefaction and KLK1, 2, 5–8, 10, 13, and 14 in individuals with SHV semen were observed [58]. In agreement with these findings, men with low concentration of seminal KLK3 have reduced sperm motility [59]. Accordingly, KLK14 levels are significantly lower in individuals with clinically delayed liquefaction and in asthenospermic infertile men [28]. In addition, targeted inhibition of KLK14 activity by the pharmacological inhibitor ACT_G9_ (based on serum KLK3 inhibitor α1-anti-chymotrypsin) in seminal plasma considerably delays semen liquefaction [28].

Gupta et al. [60] sequenced *KLK3* gene in 875 infertile and 290 fertile men and identified a total of 28 substitutions in *KLK3* coding region. Of 28 *KLK3* substitutions, 5 SNPs (rs266881, rs174776, rs1810020, rs266875, and rs35192866) appear to be strong risk factors for male infertility, while 1 SNP (c.206 + 235 T > C) is protective [60]. Variations in other KLKs have also been correlated with male infertility. Lee and Lee [61] performed genotypic association analysis in 218 non-obstructive azoospermic and 220 fertile controls and showed that a SNP in the *KLK2* intron 1 (+255 G > A, rs2664155) was associated with male infertility. Savblom et al. [62] also reported the association of SNPs in *KLK3* and *KLK2* with concentrations of KLK3 and KLK2, respectively, in seminal plasma and serum. These studies indicate that genetic variations in *KLK2* and *3* could also directly affect their enzymatic activity and hence semen liquefaction, ultimately affecting fertility.

### *SEMG* mutations

Genetic alterations of *SEMG1* variant rs147894843 is involved in altered proteolytic activity, which may affect semen quality and liquefaction leading to infertility in men [57]. In a recent study, the association between *SEMG* variants and male infertility was examined [63]. In Chinese–Han male population, the *SEMG1* variant rs2301366 was associated with abnormal semen parameters such as semen volume, sperm concentration, sperm number per ejaculate, and sperm motility and more susceptible to infertility [63]. In another study, a negative correlation between sperm motility and the proportion of SPMI (of SEMG sequence)-bound spermatozoa was also found in male subjects including infertile normozoospermics, asthenozoospermics, and oligozoospermics [12]. These findings suggest that SEMGs remained on the sperm surface post-liquefaction might account for impaired sperm motility in infertile men. In a functional proteomic analysis of seminal plasma proteins, SEMG1 isoform b pre-pro-protein levels were elevated in oligozoospermic men with abnormal sperm morphology when compared to donors with normal sperm count and morphology [64]. Interestingly, Thacker et al. [65] analyzed the major proteins in the semen from fertile and infertile men and found that infertile men lacked SEMG2 precursor showing unique differences in the semen profile. Therefore, SEMGs are crucial for liquefaction process, and mutations in *SEMGs* may have a profound impact on sperm function that goes beyond liquefaction process.

In mice, seminal vesicle secretion 2 (SVS2), an ortholog of human SEMG1, is a major seminal vesicle secreted protein that acts as a decapacitation factor and controls sperm motility [66]. SVS2 maintains sperm cholesterol levels and prevents spontaneous sperm capacitation in the uterus [67]. In the oviduct, the removal of SVS2 from the sperm’s surface induces a decrease in cholesterol from the sperm membrane, thereby resulting in the ability of sperm to fertilize the eggs [67]. *Svs2^−/−^* mice are subfertile due to copulatory plug formation defect [68]. Additionally, SVS2 has been demonstrated to protect sperm against the spermicidal uterine environment as the sperm from *Svs2^−/−^* mice were killed in the uterine cavity and failed to reach the eggs in the oviduct [68]. SVS7, also known as PATE4 (prostate and testis expression 4), is another major seminal vesicle secreted protein essential for copulatory plug formation [69]. SVS7 has been shown to enhance mouse sperm motility in vitro [70], and *Svs7^−/−^* mice exhibit subfertility due to defects in copulatory plug formation ([69], reviewed in [71]).

In addition to SEMGs and SVSs, transglutaminases (TGMs) could also potentially involve in the enzymatic complex during liquefaction. In humans, SEMGs are important substrates for TGM [72]. TGM catalyze protein crosslinking by formation of N-ε-(γ-glutamyl)lysine cross-bridges between lysine and glutamine residues of donor and acceptor proteins respectively (reviewed in [73]). TGM4 is a prostate-specific autoantigen and plays a critical role in male reproduction. TGM4 autoantibodies are detected in subfertile adult men, and these patients elicit an autosomal recessive disorder caused by mutations in the autoimmune regulator (*AIRE*) gene [74]. Accordingly, *Aire^−/−^* mice develop TGM4 autoantibodies, have compromised TGM4 secretion, prostatitis, and are subfertile [74]. In mice, the copulatory plug formation is mediated by TGM4, which catalyzes the formation of N-ε-(γ-glutamyl)lysine cross-bridges between SVS proteins ([75], reviewed in [71]). *Tgm4^−/−^* male mice are subfertile due to faulty copulatory plug formation and seminal fluid viscosity [75]. Several glutamine and lysine residues in SVS proteins 1–4 serve as substrates and are target sites for TGM4 cross-linking and may aid in copulatory plug formation, reviewed in [71]. These findings indicate that functional SEMGs, SVSs, and TGM4 are required for normal male fertility in humans and mice.

### Prostatectomy

If KLK3 is the key executor of semen liquefaction process, a loss of KLK3 production due to surgical removal of prostate glands (prostatectomy) would result in a liquefaction defect and ultimately male infertility. In patients with localized prostate cancer, radical prostatectomy is performed, which involves the removal of the entire prostate gland, the seminal vesicles, and the vas deferens. As the continuity of the genital tract is disrupted, seminal emission and ejaculation is lost (anejaculation), leading to obstructive azoospermia, but spermatogenesis normally persists in these patients. Nerve sparing radical prostatectomy may also result in erectile dysfunction [76]. Therefore, it is difficult to determine the absolute requirement of prostate-derived KLK3 in human reproduction. Or it is also possible that KLKs from other tissues (i.e., the female reproductive tract) can also contribute to the liquefaction process. This possibility will be discussed in a later section.

### Enzymatic activity of endogenous protease inhibitors from male accessory glands

Although endogenous protease inhibitors are not directly shown to be involved in semen liquefaction, studies suggest that their activity may affect functions of SEMGs and KLKs. Therefore, the importance of these endogenous protease inhibitors will be discussed briefly below.

#### Human epididymal protease inhibitor

Epididymal protease inhibitor (EPPIN) plays a critical role in sperm function and male fertility. EPPIN is a serine protease inhibitor containing both Kunitz-type and whey acidic protein (WAP)-type four disulfide core protease inhibitor consensus sequences and is expressed in testis and epididymis [77]. In the ejaculate coagulum, EPPIN is localized on the sperm surface and bound to SEMG1 (at Cys^239^) where it acts as a decapacitating factor by modulating the activity of KLK3 [10, 78, 79]. EPPIN-bound SEMG1 is critical for the degradation of SEMGs during semen liquefaction and for the initiation of progressive sperm motility in vivo [11]. *EPPIN* gene (*SPINLW1*; serine protease inhibitor-like with Kunitz and WAP domains 1) is significantly upregulated in the caput epididymis of infertile men with non-obstructive azoospermia compared to fertile patients [80]. Genetic variants of the *EPPIN* are also reported to be associated with idiopathic male infertility. In the Chinese–Han population, Ding et al. [81] reported the association of *EPPIN* variant rs2231829 with decreased risk of idiopathic infertility, while variant rs11594 increased the risk of idiopathic male infertility with abnormal semen parameters such as semen volume, sperm concentration, and sperm motility. However, there are no differences in risk for these genotypes among men with normal semen parameters, suggesting that men with different *EPPIN* variants have either an elevated or a reduced frequency of abnormal sperm parameters [81].

#### Serpin peptidase inhibitor, clade E, member 2

Serpin peptidase inhibitor, clade E, member 2 (SERPINE2) is also known as Kunitz-type or protease nexin-1 (PN1) and is highly expressed in seminal vesicles [82]. SERPINE2 has broad protease inhibitor activity against serine proteases (such as thrombin, plasminogen activators, trypsin, and plasmin) and has been demonstrated to block protein tyrosine phosphorylation and inhibit sperm capacitation [82]. Protein tyrosine phosphorylation is essential for sperm functions such as motility, capacitation, hyperactivation, acrosome reaction, and fertilization [83, 84]. *Pn1^−/−^* mice are infertile due to altered seminal protein composition, which leads to inadequate semen coagulation and deficient vaginal plug formation upon copulation [85]. On the other hand, abnormally high PN1 levels were reported in semen of men with seminal vesicle dysfunction when compared to seminal plasma from fertile men who had low PN1 levels, indicating that controlled extracellular proteolytic activity is important for fertility in humans [85]. However, it remains unclear if SERPINs are involved in the liquefaction process in mammals.

#### Serine protease inhibitor Kazal-type

Serine protease inhibitor Kazal-type 2 (SPINK2) is an acrosomal protein localized in the human mature spermatozoa [86]. Comparative gene expression profiling of infertile men diagnosed with azoospermia showed that *SPINK2* expression was decreased fourfold compared with fertile men [87]. Genetic variation of *SPINK2* is also reported to be associated with male infertility, where homozygous *SPINK2* mutation leads to azoospermia while haploinsufficiency can result in oligozoospermia [86]. In mice, SPINK2 is expressed in the germ cells and epididymis where it protects the developing sperm against protease activity during spermatogenesis [88]. *Spink2* mutant mice exhibit significantly impaired fertility accompanied by elevated serine protease activity [88], while *Spink2^−/−^* mice are azoospermic and infertile [86].

SPINK3 is a seminal vesicle-secreted protease inhibitor, which binds to the plasma membrane of the mouse sperm and appears to have protective function against protease activity in the uterus [89]. In addition, SPINK3 prevents the premature acrosomal reaction of the sperm until fertilization through reduction in endogenous nitric oxide [90].

Although the role of human SPINKs in semen liquefaction is unknown, SPINK5 is known to specifically inhibit KLK5, 7, and 14 activities in corneocytes and regulate desquamation process [91, 92]. The absence of SPINK5-mediated inhibition of KLK5, 7, and 14 is implicated in Netherton syndrome, a severe skin disorder with impaired keratinization and hair malformation [91–93]. However, it is unclear if *Spink*5^−/−^ mice are fertile [93]. Similarly, SPINK6 is a potent inhibitor of KLK2, 4, 5, 6, 7, 12, 13, and 14 and plays an important role in skin barrier homeostasis [94, 95]. In the context of semen liquefaction, preventing the activation of KLKs by SPINK5 and 6 could potentially lead to liquefaction defects.

Epididymal specific protein, SPINK13 is associated with the sperm membrane and essential for acrosomal integrity, sperm maturation, and fertility in rats [96]. A related protease inhibitor, SPINKL (SPINK-like) is another seminal vesicle-secreted protease inhibitor reported to prevent premature sperm capacitation in mice [97]. Nevertheless, the results obtained from these rodent and human studies highlight the importance of a balance between proteases and their regulation by inhibitors, which may disrupt liquefaction by suppressing protease activities of KLK in the semen.

### Endogenous proteases and protease inhibitors in the female reproductive tract

After ejaculation, semen is exposed to numerous secretory proteins (including proteinases and proteinase inhibitors) from the female reproductive tract. The distribution of KLKs in female reproductive tract varies widely (Table 2) [19, 98]. Immunohistochemical (IHC) studies revealed the expression of KLK5, 6, 11, 12, and 13 in the vaginal stratified squamous epithelium, cervical mucus-secreting epithelium, glandular epithelium of Fallopian tubes, and endometrium [98]. Additionally, in an earlier study using enzyme-linked immunosorbent assay (ELISA) performed in adult tissue, Shaw and Diamandis [19] detected the presence of multiple KLKs in the vagina (KLK1, 5–14), cervix (KLK1, 4–6, 8, 11–14), uterus (KLK1, 4, 6, 9, 11–14), Fallopian tube (KLK1, 6, 7, 9–14), and ovary (KLK1, 6–8, 10, 11, 14) at varying concentrations. Cervical–vaginal fluid (CVF) hydrates the mucosa of the vagina and ectocervix. CVF contains large amounts of both endogenous proteases and protease inhibitors [99, 100]. In the CVF, multiple KLKs (excluding KLK2, 4, and 9) are also detected using ELISA and proteomic analyses [99, 100]. The presence of KLKs in the CVF is thought to be a combined secretory action by the tissues and glands in the female reproductive tract. Secretory protein levels of KLK11–13 in the CVF are remarkably high and only exceeded by KLK2 and KLK3 levels in seminal plasma. In addition, endogenous inhibitors such as α2-macroglobulin, SERPINs, and SPINKs that regulate KLK activity have also been detected in the CVF [99, 100].

**Table 2:** Protease and protease inhibitors in the female reproductive tract.

Region	Description
Serine protease	
Vagina	KLK1, 5–14 detected by ELISA [19]
	KLK5, 6, 11, 12, 13 expression detected using IHC in the vaginal stratified squamous epithelium [98]
	Estradiol decreased KLK6, 10, and 11 levels in vaginal epithelial cells [98]
Cervix	KLK1, 4–6, 8, 11–14 detected by ELISA [19]
	KLK5, 6, 11, 12, 13 expression detected using IHC in the mucus-secreting epithelium [98]
	KLK5 and 12 cleave MUC4 and 5B in the endocervical epithelium leading to collagen remodeling [98]
	Estradiol upregulated *KLK4*, *5*, and *8* expression in ectocervical cells [101]
Uterus	KLK1, 4, 6, 9, 11–14 detected by ELISA [19]
	KLK5, 6, 11, 12, 13 expression detected using IHC in the glandular epithelial cells of the endometrium [98]
	*KLK1* expression is upregulated in endometrium during mid-menstrual cycle [102]
Fallopian tube	KLK1, 6, 7, 9–14 detected by ELISA [19]
	KLK5, 6, 11, 12, 13 expression detected using IHC in the glandular epithelium [98]
Ovary	KLK1, 6–8, 10, 11, 14 detected by ELISA [19]
CVF	KLK1, 3, 5–8, 10–15 detected using ELISA and proteomic analyses [99, 100]
	Progesterone increases KLK5–7, 11, and 12 levels in the CVF [98]
Serine protease inhibitor	
CVF	SPINK5 is specific inhibitor of KLK5, 7, and 14 activities [100]
	SPINK6 is a potent inhibitor of KLK2, 4, 5, 6, 7, 12, 13, and 14 activities
	Estradiol upregulates *SPINK5* and *SPINK6* expression in ectocervical cells [101]

In the female reproductive tract, *KLK* expression is regulated by female steroid hormones [98, 101]. Progesterone appears to stimulate KLK expression as levels of KLK5–7, 11, and 12 in CVF peaked after ovulation and positively correlated with the levels of progesterone [98]. In contrast, estradiol (E_2_) treatment decreased the concentrations of KLK6, 10, and 11 in a vaginal epithelium cell line [98], but increased *KLK4*, *5*, and *8* in ectocervical cells [101]. Moreover, *KLK1* is expressed at high concentrations in human endometrium during mid-menstrual cycle when circulating E_2_ is elevated [102]. Similarly, *Klk1* expression in mouse and rat uteri is stimulated by E_2_ [103]. In a recent study, Li et al*.* [101] reported that the expression of *KLK*s (*KLK4,5*, and *8*) and proteinase inhibitors *SPINK5* and *SPINK6* in human ectocervical cells is regulated by E_2_ in an estrogen receptor (ESR1)-dependent manner. Additionally, cell type-specific deletion of *Esr1* in the epithelial cells of the female reproductive tract (*Wnt7a^Cre/+^Esr1*^f/f^ mice) severely reduces the expression of uterine *Klk* genes (*Klk1* and *Klk1b*) [101]. Although the anatomy between human and mice reproductive tract is different, the contribution of the female factor found in the *Wnt7a^Cre/+^Esr1*^f/f^ mice provides fundamental evidence that the exposure of post-ejaculated semen to the suboptimal microenvironment in the female reproductive tract leads to faulty liquefaction and subsequently causes a fertility defect. Therefore, it is possible that an imbalance between proteases and protease inhibitors due to abnormal estrogen signaling within the female reproductive environment may disrupt liquefaction, which could be one of the reasons for unexplained infertility observed in humans.

Mucins (MUC) are the primary glycoproteins comprising cervical mucus and are thought to influence sperm transport through the cervix and uterus as they allow sperm penetrance. Apart from contributing to activation of semen liquefaction cascade, KLK5 is also responsible for digestion of collagen and modification of mucins [98]. MUC4 and 5B are the major mucins in the endocervical epithelium and are cleaved by both KLK5 and 12 in vitro [98]. Therefore, collective proteolytic action of KLKs from the seminal plasma and secretions from the female reproductive tract are crucial for normal semen liquefaction, sperm release, and transport to the site of fertilization in Fallopian tubes.

## Development of inhibitors for semen liquefaction

Current contraceptive technologies fail to meet the needs for all women. Hormonal methods of contraception, including oral contraceptive pills (OCPs), dermal patches, injections, and implants, are highly effective and reversible. However, a critical drawback of hormonal contraceptives arises from concerns over the long-term effects of hormones on patient health [104]. For instance, estrogen-containing OCPs have been linked to an increased risk of venous thrombosis [105], breast cancer [106], among other pathologies [107, 108]. Uterine bleeding is also a common reason given for women to discontinue progestin-only regimens [109]. The current over-the-counter contraceptives (condoms and spermicides) are associated with high failure rates [110]. In addition, usage of spermicides can damage vaginal and cervical mucosa increasing the risk of viral infection in women [111–113]. Therefore, there is a need for new non-hormonal vaginal contraceptives for women that can be used on demand. As mentioned above, pathophysiology, genetic inhibition, or biochemical inhibition of liquefaction process negatively impacts fertility in humans. Therefore, blocking KLK3 activity remains the prime candidate for the development of new contraceptives as it would prevent semen liquefaction and sperm transport in the female reproductive tract, potentially leading to clinical use.

**Figure 4:**
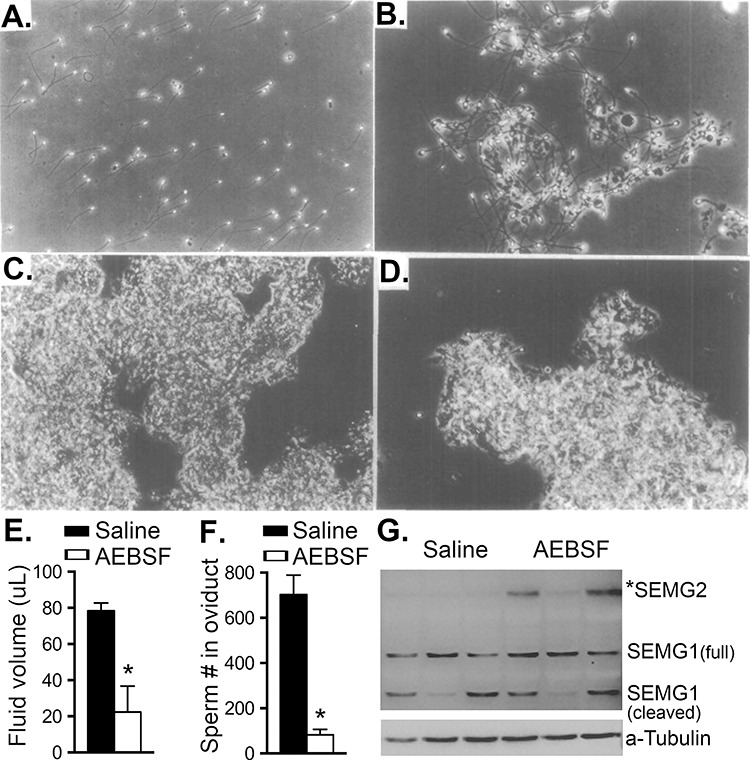
Treatment of pan-serine protease inhibitors in (A–D) human ejaculate and (E–F) female mice. (A) Human spermatozoa after liquefaction (336× magnification). Inhibition of liquefaction after Fusan treatment: (B) 1 mM Fusan (400×), (C) 10 mM Fusan (200×), (D) 10 mM Fusan (400×). (E, F) Female mice were transcervically treated with AEBSF before mating, and semen was collected ~8 h after mating. (E) Seminal volume was severely reduced in female mice treated with AEBSF compared to saline (Vehicle). (F) Total sperm number in the oviduct is significantly reduced in AEBSF-treated mice. (G) A lack of SEMG2 cleavage in the uteri from female mice treated with AEBSF. ^*^P < 0.05, unpaired *t-*test. Reprint of original (A)–(D) images with permission from [114]. Images (E, F) were modified with permission from [101] under the Creative Commons Attribution License.

Inhibition of key components regulating the coagulation and liquefaction has been previously assessed in vitro using pan-serine protease inhibitors. Early studies by Matsuda et al*.* [114] demonstrated that treatment of human ejaculates with proteinase inhibitor, Fusan (6-amidino-2-naphtyl-6-guanidinobenzoate dihydrochloride), for 30 min inhibited liquefaction (Figure 4A–D), caused solidification of semen, and completely inhibited sperm motility. Other studies focused on the use of commercially available synthetic serine protease inhibitors [such as 4-(2-aminoethyl)benzenesulfonyl fluoride (AEBSF) and phenylmethylsulfonyl fluoride (PMSF)], heavy metal cations (Zn^2+^ and Hg^2+^), and heavy metal chelator 1,10-phenanthroline to partially or completely inhibit KLK3 activity in vitro [8, 115]. However, these inhibitors have never been tested in in vivo models until recently.

Li et al. [101] showed that AEBSF effectively inhibited semen liquefaction in vivo using a mouse model as there was a lack of SEMG2 cleavage in semen collected from uterus. This semen liquefaction blockade by AEBSF treatment caused severe reduction of sperm transport to the oviduct compared to vehicle treatment in vivo (Figure 4E–G). As a proof-of-concept to determine whether protease inhibitors could potentially be used as a contraceptive, we performed a study using a pan-serine protease inhibitor (AEBSF). We showed that AEBSF (1) effectively and reversibly reduced fecundity in female mice; (2) acted as spermicide and inhibited sperm motility, resulting in a decreased fertilization in vivo and in vitro; and (3) was significantly less damaging to the vaginal epithelium (compared to N9) when treated for 10 min or three consecutive days in vivo in mice [116]. This review is preceding the report on our AEBSF study in this Special Issue. Despite inhibitory capacity of AEBSF, their application as therapeutic agent is hampered due to a lack of selectivity. Therefore, increased interest for development of a highly potent and selective inhibitor toward KLK3 activity that would cause blockade of semen liquefaction and sperm transport within the female reproductive tract will have potential pharmaceutical utility as a novel contraceptive.

**Figure 5:**
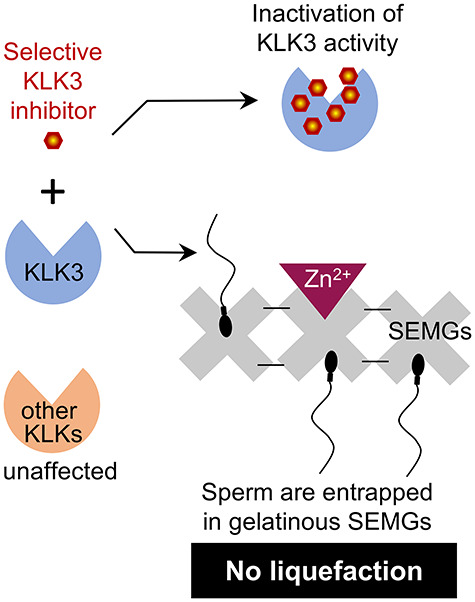
Using of specific KLK3 inhibitors as a novel contraceptive method to block semen liquefaction process. KLK3 inhibitor will potentially attenuate semen liquefaction process as its activity will be specific to KLK3 and does not affect other KLKs or other serine proteases in semen as well as in the female reproductive tract.

Before moving on to the selective inhibitor for KLK3, it is important to note that anti-EPPIN was also developed as a male contraceptive; however, the goal was to decrease sperm motility and not inhibition of the semen liquefaction. Briefly, a study in *Macaca radiata* monkeys immunized with recombinant human EPPIN showed an effective and reversible male infertility without hormone disruption [117]. Treatment of human spermatozoa with anti-EPPIN antibodies inhibited EPPIN–SEMG1 interaction and significantly decreased sperm motility [11]. Furthermore, anti-EPPIN antibodies have been demonstrated to inhibit human sperm acrosomal reaction, reduce intracellular Ca^2+^ concentration, and does not alter tyrosine phosphorylation of sperm proteins [118]. The use of sperm surface EPPIN as a non-hormonal contraceptive target has led to the development of small organic compounds that could substitute for SEMG1 or anti-EPPIN antibodies and provide a reversible, short-lived pharmacological alternative. In this regard, EP055 is a 1,3,5-triazine compound that targets EPPIN on the surface of sperm and inhibits motility [119]. Intravenous infusion of EP055 in male macaques demonstrated plasma half-life of 11 min and the drug being retained in semen for up to 78 h followed by recovery of sperm motility [119]. Although EPPIN modulates the hydrolysis of SEMGs by KLK3, it is not an effective inhibitor of KLK3 activity [120, 121]. Therefore, mode of action of anti-EPPIN will be different than that of semen liquefaction inhibition.

## Development of small molecule(s) inhibitors specifically for KLK3

KLK3 is an ideal target for the development of small-molecule inhibitors targeting its enzymatic activity that would allow development of non-invasive contraception technologies (Figure 5). Most of the studies involved in generation of small molecule inhibitors of KLK3 were focused on their usage in targeted treatment of prostate cancer (Table 3). The first KLK3 inhibitors reported in the literature used a homology model derived from porcine KLK to design and synthesize β-lactam analogs, which showed promising inhibitory activity with an IC_50_ (inhibitor maximal inhibitory concentration) as low as 226 nM [122]. To obtain mechanistic insights into the inhibition of KLK3, Singh et al. [123, 124] showed that β-lactam based inhibitors compete with KLK3 substrates and form a stable covalent complex at the catalytic Ser^189^ residue in a time-dependent manner. Other strategies include the use of azapeptides, which target both cysteine and serine proteases and effectively inhibit KLK3 activity with the K_i_ (inhibition constant) as low as 500 nM [125].

Using high-throughput screening of chemical libraries, Koistinen et al. [126] screened 49 920 compounds to identify small drug-like molecules and pinpointed two compounds inhibiting KLK3-activity in a dose-dependent manner in human umbilical vein endothelial cells [126]. These two active compounds contain either benzoxazinone or triazole derivatives and exhibit potent KLK3 inhibition with IC_50_ of 300–500 nM but lack selectivity toward KLK3 activity [126]. In addition, triazole derivatives have also been identified to inhibit other KLKs such as KLK5, 7, and 14, as well as matriptase (a transmembrane serine protease) [127]. Therefore, the non-selective mechanism of serine protease inhibition by β-lactam analogs and azapeptides severely limited their development as therapeutic drugs as they possess off-target inhibitory activity.

LeBeau et al. generated a series of small-molecule active-site inhibitors of KLK3 using peptide aldehydes and boronic acid-based inhibitors containing Ser-Ser-Lys-Leu-Gln peptide present in SEMG2 (a natural specific substrate for KLK3) as a template. Peptide aldehyde inhibitors showed specific proteolytic activity toward KLK3 with more than 20-fold specificity than that of chymotrypsin but had a K_i_ of 6.5 μM [128]. Boronic acid modification led to the development of more potent inhibitors with high specificity and a K_i_ of 65 nM, 100-fold lower than the aldehyde-modified peptide inhibitors [128]. In vivo evaluation of this inhibitor through intravenous injection at a dose of 33 mg/kg for two cycles of three consecutive days in human prostate cancer xeno-grafted mice led to significant reduction in KLK3 serum levels (free KLK3 levels by 35% and total KLK3 levels by 30%), however, had minimal effect on tumor growth [128]. Subsequent modification of this inhibitor by introducing the non-natural amino acid nor-leucine (Nle) exhibited potent KLK3 inhibition with a K_i_ of 48 nM [129].

**Table 3:** Summary of key KLK3 inhibitors reported in the literature.

Type	Relevance	Agent	Description/pharmacological data/therapeutic impact
β-lactam analogs	Unclear	2-azetidinone	IC_50_ = 226 nM [122]
	Prostate cancer	Benzoxazinone derivatives	K_i_ = 300 nM. 30 times more selective compared to chymotrypsin (K_i_ = 8.5 μM) [126]
		Triazole derivatives	K_i_ = 500 nM. 10 times more selective compared to chymotrypsin (K_i_ = 5.4 μM) [126]
Cysteine and serine protease inhibitors	Prostate cancer	Azapeptides	K_i_ = 500 nM [125]
Heavy metal cations	Semen liquefaction	Zn^2+^	Inhibits KLK3 activity at 10 mM [8]
			IC_50_ = 20 μM [41]
		Hg^2+^	Inhibits KLK3 activity at 10 mM [8]
			IC_50_ = 150 μM [41]
		Cu^2+^	IC_50_ = 150 μM
		Cd^2+^	IC_50_ = 200 μM
		Co^2+^	IC_50_ = 500 μM
Heavy metal chelator	Semen liquefaction	1,10-phenanthroline	Inhibits KLK3 activity at 50 mM [8]
Pan-serine protease inhibitors	Semen liquefaction	PMSF	Inhibits KLK3 activity at 5 mM [8]
		AEBSF	Inhibits KLK3 activity at 5 mM [8]
	Prostate cancer	PMSF	Inhibits KLK3 activity at 20 mM [115]
		AEBSF	Inhibits KLK3 activity at 10 mM [115]
Peptide aldehyde inhibitor	Prostate cancer	Z-SSKLL-H	K_i_ = 6.5 μM [128]
Peptidyl boronic acid inhibitor	Prostate cancer	Z-SSKL(boro)L	K_i_ = 65 nM. 60 times more selective compared to chymotrypsin (K_i_ = 3.9 μM). Reduction in free and total KLK3 serum levels in human prostate cancer xenografts produced in nude mice upon intravenous administration of 33 mg/kg dose for two cycles of three consecutive days/5 days [128]
		Z-SSKn(boro)L	K_i_ = 48.4 nM. Norleucine substitution of Z-SSKL(boro)L [129]
		Ahx-FSQn(boro)Bpg	K_i_ = 72 nM. Eight times more selective compared to chymotrypsin (K_i_ = 580 nM). Reduction in free and total KLK3 serum levels in human prostate cancer xenografts produced in nude mice upon intravenous administration of 10 mg/kg dose for three cycles of five consecutive days/week [130]
RNA aptamer	Prostate cancer	Not applicable	Synthetic RNA molecules (92 mer) selected from pools of random-sequence oligonucleotides to specifically bind active KLK3 [131]

To enhance selectivity of KLK3, Kostova et al. generated a peptidyl boronic acid-based selective KLK3 inhibitor containing a bromopropylglycine group, which had a K_i_ of 72 nM and eightfold selectivity over chymotrypsin. Systemic administration of this compound at a dose of 10 mg/kg for three cycles of five consecutive days in nude mice with human prostate cancer xenografts showed minimal effect on tumor growth but led to significant reduction in serum KLK3 levels [130]. Other novel KLK3-targeting therapeutic strategies involve the use of RNA aptamers, synthetic nucleic acid molecules, selected from pools of random oligonucleotides via the systematic evolution of ligands by exponential enrichment (SELEX) process to specifically target active KLK3 [131]. However, the selectivity of RNA aptamers on KLK3 activity has not yet been tested in vivo*.*

To date, numerous endogenous inhibitors of KLK3 activity with physiological significance ranging from metal ions (Zn^2+^) to proteinase inhibitors (SERPINs) have been reported, but none have been employed for the development of contraceptives to inhibit semen liquefaction in vivo. Additionally, the development of selective KLK3 inhibitors was focused for targeted treatment of prostate cancer. Although numerous small molecule and peptides to inhibit KLK3 activity have been developed, they may not be suitable for contraceptive purposes because the activity is not entirely specific to KLK3 but also bind to a great variety of proteases. An unusual feature of SERPINs is their ability to often inhibit non-target cysteine proteases, i.e., cross-class inhibition [132]. In addition, peptide inhibitors are often pH-dependent, thus may not withstand the relatively low pH in vaginal microenvironment [133].

## Conclusion

Human semen liquefaction is a post-ejaculation proteolytic process that changes semen from a gel-like coagulum to a watery consistency (liquefied) and is mainly governed by SEMGs and prostate-derived KLK enzymatic activities. The blockade of semen liquefaction prevents sperm migration in the female reproductive tract and is an unexplored target for both male and female contraception. Inhibition of semen liquefaction can be achieved by using molecules that can stabilize SEMGs (preventing hydrolysis), local delivery of exogenous metal ions (Zn^2+^), overexpression of endogenous protease inhibitors (SERPINs/SPINKs), or administration of synthetic serine protease inhibitors. Of the numerous key molecules involved in the liquefaction cascade, targeting KLK activities (i.e., KLK2, 3, 5, and 14) is a viable option due to the fact that these KLKs are produced specifically in the prostate gland, hence, providing a localized target for the development of a non-hormonal contraceptive.

One of the immediate possibilities is the use of specific KLK3 inhibitors that were previously developed for prostate cancer patients. KLK3 in the seminal plasma is secreted at extremely high concentration, relative to other KLKs, and is the key executor enzyme involved in semen liquefaction. Rather than using a pan inhibitor of KLK gene family, which could potentially lead to non-intended effects, studies focusing on the development of small drug-like molecules specifically inhibiting seminal KLK3 activity would prove useful in the development of novel non-steroidal, over-the-counter contraceptive with improved efficiency.

## Conflict of interest

The authors have declared that no conflict of interest exists.
